# Designing for Amplifying Voices—Supporting Bangladesh’s Low-Income Ready-Made Garment Workers Through Technology Amid COVID-19 Pandemic: Interview Study Among Workers

**DOI:** 10.2196/90675

**Published:** 2026-10-02

**Authors:** Rahat Jahangir Rony, Nova Ahmed

**Affiliations:** 1 School of Computer Science and Informatics, Cardiff University, Abacws, Senghennydd Road, Cardiff, Wales, CF24 4AG, United Kingdom, +44 7311249074; 2 Department of Electrical and Computer Engineering, Design Inclusion and Access Lab (DIAL), North South University, Dhaka, Bangladesh

**Keywords:** ready-made garment workers, RMG, COVID-19 pandemic, well-being, technology use, health care, Bangladesh, occupational health, digital literacy, health management, technology-based, framework

## Abstract

**Background:**

The ready-made garments (RMG) industry is a crucial component of Bangladesh’s economy, using over 4 million workers from low-income backgrounds who often neglect their health care needs. Historically, this sector has faced criticism for labor exploitation, unsafe working conditions, and rights violations, as highlighted by tragic accidents resulting in loss of life. Compliant factories may uphold higher labor standards, but many noncompliant factories expose workers to poor conditions, increasing health risks. The COVID-19 pandemic intensified vulnerabilities, leading to widespread factory closures and job losses, increased health risks, and left millions of workers without wages. Although the government attempted to provide some relief, it fell short in offering job security, social protection, health services, and emergency assistance. Limited access to technology due to digital literacy gaps further hinders these workers, who primarily use basic mobile phones for communication rather than accessing health or emergency services. Thus, there is a pressing need to develop a sustainable system that capitalizes on their existing technological familiarity.

**Objective:**

Our goal was to gain a deep understanding of RMG workers’ experiences, focusing on their work environments, technological interactions related to health care management, and the impacts of COVID-19 on their circumstances. This understanding aims to inform the design of a technology-based framework that is both sustainable and contextual.

**Methods:**

In phase 1, we conducted in-person interviews with 55 RMG workers, comprising 32 female and 23 male participants from urban and suburban areas of Dhaka and Gazipur, before the pandemic. Participants were aged between 18 and 40 years. In phase 2, we reconnect with 12 phase 1 participants during the pandemic and also consulted 3 stakeholders from RMG factories via phone. Each interview, conducted in Bengali, was recorded with consent, totaling 846 minutes of discussion that were translated and transcribed. Thematic analysis was used to analyze the results.

**Results:**

Insights gathered revealed variations in working conditions, personal experiences, perceptions of health care, lifestyle choices, and technology use tied to factory compliance. Workers at compliant factories had better health care support and used technology more effectively than those in noncompliant settings. The pandemic drastically altered the landscape for all workers, heightening health and safety concerns and shortages of emergency assistance. The RMG sector faces significant challenges, highlighting the urgent need for targeted emergency relief and health services.

**Conclusions:**

This research examined the challenges and technology use among RMG workers during the pandemic, with a focus on health care perspectives. Based on our findings, we proposed a technology-based framework called VOICE (Virtual Outlet for Integrated Community Engagement) that connects workers with service providers through a simplified interface. This aims to assist marginalized communities during emergencies and enhance their overall well-being.

## Introduction

### Background

The ready-made garment (RMG) industry is a crucial sector of Bangladesh’s economy, contributing over 10% to the national gross domestic product and 84% of total export earnings [1-3]. The sector employs over 4 million workers, with nearly 90% being female workers from low-income and rural backgrounds [4,5]. This aligns with Lewis’s economic growth model, characterized by an unlimited labor supply driven by low labor costs [4,6]. As of 2018, the entry-level wages for workers were US $62.42 [7-10]. The government of Bangladesh raised monthly wages by about 51% that year, setting the minimum wage at $94.22 [10]. In 2023, the entry-level wage was adjusted to BDT 12,500 ($102) per month with a 9% annual increment [11], which still reflects low pay. Though this sector is economically significant, it has also been criticized for labor exploitation, unsafe working conditions, and violations of workers’ rights [12-15]. Such incidents have sparked greater awareness of workers’ rights, mandating that all RMG factories comply with internationally recognized standards [14,16-19]. In Bangladesh, RMG factories are divided into compliant and noncompliant factories. While some facilities follow international labor standards and provide workers with better rights and living conditions (compliant factories) [20], many (noncompliant factories) expose workers, especially female, to verbal and physical abuse, lack of sanitation, long working hours, excessive workloads, and health risks [20-24]. Such a situation impacts the health of workers, especially female workers. Accordingly, health care awareness among RMG workers remains low [25], and access to formal health services is limited due to economic constraints, stigma, and limited health information [24,26].

The health-wealth gradient in labor markets demonstrates that workforce participation sometimes depends on workers’ capacity to accept positions, which is shaped by population health and social safety nets [27]. The COVID-19 pandemic intensified the vulnerabilities of this workforce. Global supply chain disruptions and order cancellations (approximately USD $2.8 billion worth of orders were withdrawn) by Western brands led to the closure of thousands of factories [28,29], leaving over a million workers temporarily unemployed without wages or severance pay [30]. While the government of Bangladesh attempted relief packages (approximately US $13,549 million), there were no safeguarding policies to ensure job and health security or social protection [31]. In addition, living in densely populated settlements, RMG workers faced heightened health risks, food insecurity, inadequate emergency aid, and emotional distress during the pandemic [32,33].

The pandemic has, in addition, underscored the growing role of technology in emergency response. In both developed and developing contexts, crisis response technologies, such as crowdsourcing platforms, helplines, and mobile health services, have improved access to timely support [34-36]. In Bangladesh, services such as 999 (general emergency) and 333 (COVID-19), as well as telehealth platforms such as Tonic (Telenor Health and Grameenphone) and Doctime (Doctime Limited), became available [37-39]. Recent work on AI-based risk assessment tools highlights critical considerations for deploying conversational health technologies in real-world settings, including the need for evidence of grounding and safety-focused evaluation [40]. However, these solutions often remain inaccessible to low-income populations due to limited digital literacy, lack of trust, and gendered disparities in technology use [41,42]. Workers in informal economies, including RMG workers, typically use basic mobile phones primarily without internet connectivity for communication rather than accessing health or emergency services [43,44].

Given this context, there was a critical need to understand how RMG workers interact with technology, manage health needs, and cope with crises in order to design meaningful, accessible digital interventions. Yet, no such research has explored the living realities of this particular industrial low-income group in developing countries, especially during global crises such as COVID-19.

To address this gap, we examined the daily experiences of RMG workers before and during the pandemic on the basis of compliant and noncompliant factories, with a focus on health perceptions and technology use. Specifically, we investigated:

- Research question 1: How do RMG workers manage their daily lives, health care needs, and technology use in their existing work environment*?*
- Research question 2: How did the COVID-19 pandemic affect their daily lives, health, and access to support?

### Aims and Approach

This research aimed to investigate key research questions and develop technology-based design implications that can provide emergency support for ready-made garment workers and other low-income groups during times of crisis. The goal is to create a platform that amplifies their voices. However, many existing technology services are underused due to usability barriers and a lack of contextual relevance. To address this issue, we propose a technology-based framework called VOICE (Virtual Outlet for Integrated Community Engagement). This framework leverages service interface platforms to offer emergency support while ensuring that the voices of low-income communities are heard. In this paper, we report the results of in-depth interviews conducted with RMG workers in Bangladesh.

## Methods

### Overview

We conducted qualitative interviews in 2 phases: before the COVID-19 pandemic (phase 1) and during the pandemic (phase 2) in Bangladesh, specifically between January and April 2020.

Phase 1 occurred from January to February 2020, before the COVID-19 lockdown in Bangladesh that started at the end of March 2020 (by that time, China halted the shipping of raw materials, accessories, machinery, and spare parts; it affected the apparel and textile industries [29]). During this phase, we interviewed 55 RMG workers (32 female and 23 male workers) across 6 focus group discussions (FGDs) to gain insights into their lives and daily experiences.

In phase 2, we conducted one-on-one follow-up phone interviews with 12 RMG workers (9 female and 3 male workers) from phase 1 during the first week of April 2020, after the COVID-19 lockdown was imposed in Bangladesh. Additionally, we interviewed 3 stakeholders (including merchandisers, managers, and factory owners) connected to the financial policies of the RMG sector.

### Phase 1 Method

#### Participant Recruitment Technique

We recruited participants from 3 areas in the Dhaka division: Mirpur, Ashulia, and Gazipur, as shown in Tables 1 and 2. Key informants (KIs), such as landlords and house managers who are well-known and trusted within the local community, assisted us in recruiting workers.

**Table 1.** Dimensions of research and details of the participants.

Phase 1: study before the COVID-19 pandemic (January 20 to February 20)						
FGD^a^ groups	Participants count, n	Sex	Age range (years)	Area	Factories workers are engaged, n	Working experiences (years)
Group 1	11	Female	22‐38	Mirpur, Dhaka, Urban	9	3‐10
Group 2	7	Male	25‐40	Mirpur, Dhaka, Urban	7	3‐10
Group 3	12	Female	20‐30	Ashulia, Dhaka, Suburban	3	3‐5
Group 4	8	Male	18‐30	Ashulia, Dhaka, Suburban	7	3‐10
Group 5	9	Female	18‐40	Gazipur Circle, Gazipur, Suburban	7	3‐5
Group 6	8	Male	18‐40	Gazipur Circle, Gazipur, Suburban	7	3‐15

a FGD: focus group discussion.

**Table 2.** Details of the participants.

Phase 2: study during the COVID-19 pandemic (first week of April 20)						
One to one interview	Participants count, n	Sex	Age range (years)	Area	Factories workers or stakeholders are engaged, n	Working experiences (years)
RMG^a^ workers	12	Male and Female	18‐35	Mirpur, Ashulia, and Gazipur Circle, Dhaka	10	3‐10
Factory owner	1	Female	18‐35	Dhaka	1	8
Assistant manager (compliance)	1	Male	18‐35	Dhaka	1	8
Merchandiser	1	Male	18‐35	Dhaka	1	5

a RMG: ready-made garment.

These workers frequently communicate with KIs regarding rent-related matters. The KIs managed RMG workers in various roles, such as helpers and operators, from both compliant and noncompliant factories. The participants were related but not family, such as friends and colleagues. We ensured that male and female workers were recruited separately in each area for their comfort. The participants’ age range was 18 to 40 (mean 27.37, SD 5.65) years, and they represented 40 different RMG factories, as indicated in Table 1. Among them, 31% (17/55) worked in compliant factories, while 69% (38/55) were employed in noncompliant factories. The participants were eager to share their experiences, reflecting Bangladesh’s collectivist societal structure [45]. FGDs allow participants to express their beliefs, experiences, perceptions, and attitudes [46]. Each discussion format involved an interactive exchange between participants and the research team in a group setting [46,47].

Workers typically work 6 days a week and spend their weekends, particularly Fridays, attending to necessary tasks at home and outside. To accommodate their schedules, we preferred to form large focus groups of up to 12 workers at a time within the same community. This format also encouraged participants to have their voices heard [48].

#### Study Design and Procedure

The study aimed to gain a brief understanding of the personal lives, social aspects, relationships, and everyday workplace experiences of RMG workers. We divided the interviews into 2 segments that highlight these workers’ positive and negative insights. First, we examined the daily work systems, including workload, health issues, work environment, relationships with coworkers, and interactions with supervisors. Second, we explored the workers’ personal lives, focusing on financial stability, family dynamics, friendships, sources of entertainment, technology use, social relationships, and their responsibilities regarding children.

Before each discussion, we engaged with the participants to arrange the discussion venue, prioritizing their comfort [49], and an evaluator for the qualitative study. We aimed to create an interactive zone through informal discussions. We explained the research objectives, methods, ethics, and incentives at the beginning of each discussion. We ensured participants provided consent and addressed their concerns about the topics. Once consent was granted, the lead researcher (RJR) guided the discussions, while other team members assisted with follow-up questions and took notes. The study was semistructured and conducted in Bengali, with nontechnical questions for the participants. Each FGD lasted, on average, 120 (mean 121, SD 2.19) minutes. We audio-recorded each discussion with the participants’ consent.

#### Analysis

The interviews were conducted in Bengali, which required transcription and translation of all the interviews. A total of 726 minutes of audio was translated and transcribed into English. Being fluent in both English and Bengali, we carefully transcribed and translated the audio directly into English (forward translation). We revised the translated transcription to ensure data validity and manually analyzed it. We read and reread all the transcriptions multiple times to become thoroughly familiar with the data. We started the analysis process after completing the first FGD because the collected contents and the analysis mutually apprised each other [50]. We used thematic analysis, starting with an open-inductive coding procedure that also includes both inductive and deductive processes [51,52]. After the first phase of open coding, we generated a codebook that the authors (RJR and NA) collectively revised to group similar codes into second-order codes. The first author (RJR) reviewed all the themes multiple times, and the authors (RJR and NA) generated the themes collaboratively. Theme 1 addressed the challenges RMG workers face in noncompliant factories, significantly affecting their well-being. In contrast, theme 2 emphasized the opportunities for RMG workers in compliant factories, positively impacting their quality of life.

#### Ethical Considerations

The institutional review board approved the research (application: IRB-No.0501). Participants had the right to skip any questions or withdraw from the discussions at any point without losing their incentives. All recordings, notes, and data collected are securely stored in a private drive, with access granted only to the researchers. For participating in the FGDs, we offered snacks and daily grocery items worth BDT 500 (US $4 as of Sep 1, 2026) to each participant as incentives. Each KI received the same snacks, daily grocery items, and BDT 1000 (US $8 as of Sep 1, 2026) for their additional fieldwork.

### Phase 2 Method

#### Participant Recruitment Technique and Study Design

We could reach 12 individual RMG workers (9 female and 3 male workers) from the participants of phase 1 for one-on-one phone interviews (Table 1), having collected their phone numbers during that study. The significant reduction from 55 participants in phase 1 to 12 in phase 2 reflects the extraordinary circumstances during the initial COVID-19 lockdown in Bangladesh, when participants could be reached only by phone due to a complete shutdown. This represents a subset that may not fully capture the diversity of experiences across all factory types and locations. However, they did represent 10 different factories and the same 3 geographic areas, providing some breadth.

This follow-up investigation aimed to understand the impact of the COVID-19 pandemic on these workers, including how they receive immediate support and the health and safety situations in their localities. Additionally, we formally requested that factory authorities assist us in contacting potential stakeholders in the RMG sector. Through this process, we recruited 3 participants: a factory manager, a merchandiser, and a factory owner, all from different garment factories. This allowed us to gain insight into the overall state of the RMG sectors and the future challenges workers face, buyers’ perspectives, and the role of authorities in relation to COVID-19.

#### Study Procedure and Analysis

We conducted audio phone conversations with the workers, each call lasting around 10 minutes. In total, we had 120 minutes of conversation (mean 10.1, SD 1.40 minutes). The 3 conversations with stakeholders were approximately 30 minutes each, resulting in 90 minutes of discussion (mean 30, SD 2 minutes). We did not record any phone calls and took real-time notes during the conversations. All discussions were conducted in their native Bengali language, and the lead researchers moderated the data collection.

We used the same content analysis method as previously discussed. Through inductive open coding, we generated first-order codes, grouped similar codes into second-order codes, and ultimately developed the final theme 3, which addressed how the COVID-19 pandemic made the RMG workers’ lives difficult.

#### Ethical Considerations

This study was conducted under the same institutional review board guidelines. We ensured the anonymity of all participants and securely stored the notes in a locked drive. No incentives were provided for participation in the phone conversations; we verbally thanked each participant after our calls.

## Results

### Overview

The holistic demography is shown in Figure 1. These participating workers came from the southern (19/55, 35%) and northern (36/55, 65%) regions of Bangladesh, both of which are low-income areas. Due to economic constraints, 92% (51/55) of these individuals did not complete their school-level education. Despite their limited education, many secured early employment in RMG factories, as these facilities often prioritize hiring younger, physically fit workers to boost productivity. It was interesting since, in Bangladesh, male workers generally earn more than female workers in households [53]; however, female workers contribute significantly to family earnings, particularly in the RMG sector.

**Figure 1.**
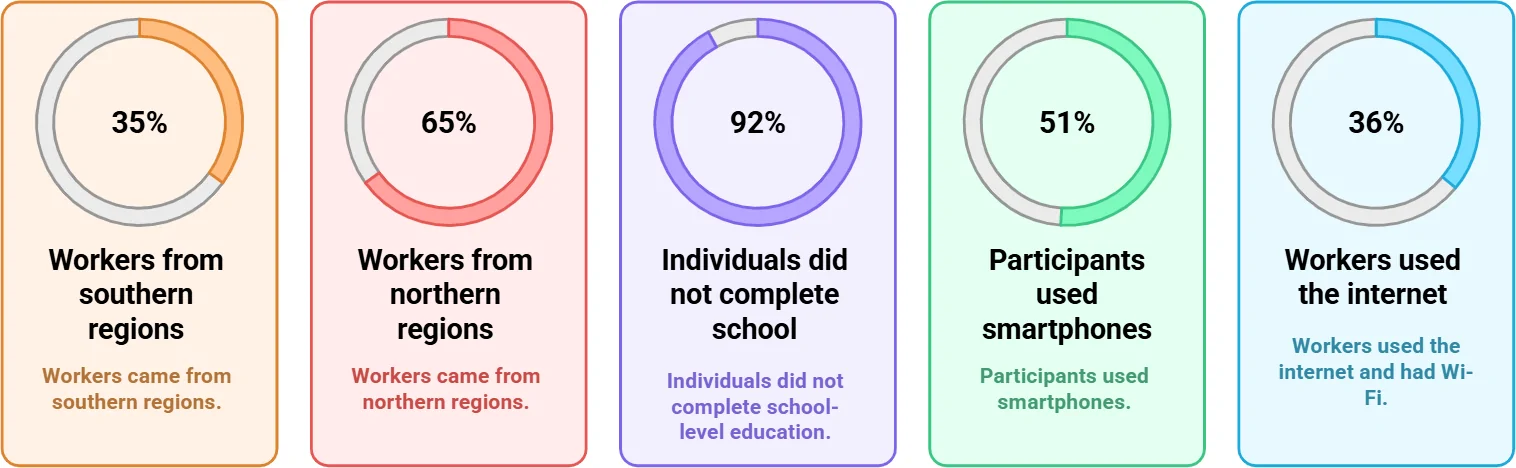
Participants' profile.

Additionally, 51% (28/55) of participants used smartphones, with higher use among male participants, though many were unaware of how to use them. About 36% (20/55) of workers used the internet and had Wi-Fi support at home, mostly from compliant factories. Their internet use was primarily limited to online calling apps (eg, IMO; PageBites, Inc) for communication with distant relatives and coworkers. A few male workers had social media but were inactive, while others rarely watched YouTube.

In the context of wages, at the time of phase 1 data collection (January-February 2020), the minimum wage for RMG workers was BDT 8000 (approximately US $65 as of August 17, 2026). However, a significant portion of workers, particularly in noncompliant factories, earn below the minimum wage. By the time of phase 2 (April 2020), the COVID-19 pandemic had led to widespread factory closures, leaving many workers without any wages or severance pay, compounding their existing financial vulnerability.

### Theme 1: Sounds of Distress From Noncompliant Factory Workers

The working environment for employees in noncompliant factories greatly affects their well-being and lifestyles. Participants noted that noncompliant RMG factories often neglect workers’ rights and health, resulting in these workers facing the harshest conditions (eg, limiting water intake or the ability to visit the toilet to increase productivity). Many female workers in noncompliant factories experience abuse. They reported that senior officers, such as line chiefs, floor managers, and supervisors, often exert pressure and punish primarily female employees. Additionally, these workers frequently do not receive their rightful salaries and remain silent about these injustices. Fearful of losing their jobs, female workers are reluctant to confront authority regarding abuse, making their situation even more challenging. Despite these hardships, they continue working due to their obligations, which impact their well-being all over. One female worker from a noncompliant factory expressed her emotional and mental state as follows:

*Some line chiefs treat us very poorly. Supervisors are mean. I frequently feel like crying–we can’t even wipe our eyes. If we cry, they will punish us by standing in front of the chamber for hours. They say this is not a place for crying; this is a workplace.*

[Female worker, Ashulia, group 3]

Male participants shared that factory authorities often prefer hiring female workers over male workers because female workers tend to be quieter and less likely to protest against irregularities and misbehavior. Many female participants agreed, mentioning that there have been fewer protests in factories due to the high number of female employees, which has led to increased injustices impacting passively their well-being. A male worker from a noncompliant factory described the biased recruitment process, stating that young, attractive, and healthy workers are favored, particularly female workers. He said:

*RMG authorities do not prefer elderly people or men. Overweight will not get a job. It’s all about being young and slim. They prefer female because they are less likely to protest against mistreatment. They can pay female less.*

[Male worker, Mirpur, group 2]

Because of such conditions, workers get sick and also often neglect their health and personal care to focus on their job responsibilities. They believe that their roles require them to remain healthy, but typically they do not receive adequate health care support from their factories. Additionally, authorities overlook workers’ health care needs and treatment costs. Many workers reported that they often do not have proper restroom breaks due to excessive pressure, which limits their water intake throughout the day. Consequently, many are concerned about potential job loss that may arise from significant health issues. One female participant highlighted how their workload adversely affects their health:

*We start working in the morning and continue without breaks, only pausing for lunch, then sitting again until we finish. We don’t drink water or go to the restroom. Drinking water makes us feel pressured to use the washroom, which hampers production. Taking bathroom breaks is not prohibited, but we avoid them saving time.*

[Female worker, Ashulia, group 3]

In addition, these workers often restrict the use of phones in the workplace. They have shared that they secretly use their phones on staircases or near restrooms to make calls during work hours. Otherwise, they will be punished. Furthermore, many do not have internet access to their home phones, as they cannot bear the additional costs of the internet for low pay. Additionally, due to heavy workload, they cannot manage time to use the phone, resulting in they cannot explore entertainment and any support options through phone. Therefore, this theme shows that noncompliant factory environments and lack of support decrease workers’ quality of life.

### Theme 2: Workers in Compliant Factories Lead Better Quality of Life

Participants from compliant factories unanimously highlighted the importance of timely salary payments and fair overtime compensation. Female workers reported significantly better job experiences, positively impacting their lives.

They also receive support from the factories regarding any workers’ issues. One female worker expressed her feelings about increased income and teamwork at compliant facilities:

*We do not feel overwhelmed by our workload. If someone is under too much pressure, management assigns additional workers to help. Sometimes, they implement a double process to meet targets. During shipment, we may work overtime until a maximum of 7 PM, and they compensate us properly for that. All the workers cooperate during shipment, and we are okay with it.*

[Female worker, Gazipur, group 5]

Participants mentioned that compliant factories provide personal health facilities. Workers can take breaks whenever needed and access plenty of drinking water during their shifts. One female participant shared that there are medical staff at the factories, which many workers rely on due to the free services provided:

*There is a medical team inside the factory. If someone feels ill, they prescribe medication, sometimes even provide medicines. They can be granted emergency leave or transported to a hospital by ambulance, with the company covering the expenses. Their salary will not be deducted; we depend on this support.*

[Female worker, Gazipur, group 5]

Compliant factories also do not impose strict restrictions on communication, providing dedicated mobile phones on the factory floors for emergency calls, which the workers feel is good, as it helps to reduce the cost of regular personal communication. One female worker explained:

*We do not use our personal mobile phones in the garments, but we are not bothered because several phones are available on every floor. We can use those for communication. If someone needs to reach us, they can call on those phones. We can talk even under heavy work pressure and frequently access them without any cost.*

[Female worker, Gazipur, group 5]

Workers with internet access at home are typically employed in compliant factories. They usually share internet costs and live in relatively better areas than workers in noncompliant factories. This support often extends beyond shared costs, primarily provided by the house owner. Some believed that internet-based technology provided better resources for their children at home. They encourage their children to use mobile phones and the internet for educational purposes. One participant shared that he bought a computer for her daughter to use it:

*She reads the Bengali newspaper online (on laptop). I encourage her to watch animations and tutorials on YouTube. I want to make my daughter a computer engineer.*

[Male worker, Ashulia, group 4]

Therefore, the theme highlighted that compliant factory workers received significantly better support at the workplace, which encouraged them to lead a quality life.

### Theme 3: COVID-19 Pandemic Makes Workers' Lives Difficult and Threatening

The COVID-19 pandemic has negatively impacted all factory workers, regardless of their compliance status. We found workers faced significant health care risks during the pandemic, as COVID-19 was highly contagious. Many workers lived in congested areas, often sharing a single floor with 6 to 8 families [54,55]. As a result, they struggled to maintain social distance, as their families shared kitchens and washrooms. Participants noted that they could not prevent their children from playing together in the evenings. This situation was consistent across all study areas. Compliant factory workers typically relied on medical support from their factories, but this support was halted due to the pandemic, raising concerns for their well-being. A female worker expressed her worries about social distancing:

*We can’t maintain social distancing because our area is very congested. If you want to do it personally, I think you can’t. We have a shared kitchen. If anyone is infected here, the situation will be dangerous. We do not have health care support or food from anywhere.*

[Female worker, Mirpur, Dhaka]

Participants reported that their factories closed suddenly, often without prior notice, and many did not receive their salaries. This lack of communication left them struggling to survive in the following days. They expressed the need to be financially prepared due to the uncertainty brought about by the pandemic, particularly concerning their ability to pay monthly house rent, grocery bills, and utility costs. Most of them found it difficult to save money due to low incomes, with many workers indicating that they had no savings to cover their monthly expenses. Some participants noted that they did not receive emergency relief from the private or government sectors since relief efforts often focused on the extremely poor. Other participants echoed a similar sentiment. This lack of adequate support was voiced by a male worker, who said:

*We do not get any salary in advance. We received no relief from anywhere, not even from our garments. The situation is the same for everyone. Our living areas are not slum areas, so government and private relief efforts focus on slum areas. The government announces salaries for workers. That might be another reason we are not receiving emergency relief. We are on an unpredictable journey. We just need food and treatment now, nothing else. We only need rice, red lentils, and oil, and that’s it.*

[Male worker, Mirpur, Dhaka]

Accordingly, the stakeholders in this study expressed concerns about the RMG sector, which has historically faced significant losses. During the study period, they noted that the Bangladesh government announced a US $592 million incentive package to support RMG factories and their workers [31]. This support is provided to 4000 registered RMG factories by the Bangladesh Garment Manufacturers and Exporters Association, all of which are either compliant or partially compliant with regulations. However, stakeholders were worried that the thousands of unregistered and noncompliant factories and their workers would not receive any incentive support. The factories lost ongoing contracts, which added financial pressure due to their reliance on imported goods from China during the early days of the COVID-19 outbreak. One support authority representative shared his thoughts on the challenges faced by RMG workers:

*The pandemic leads to food crises. People need food, treatment, not clothing. Our foreign income will decline, and we must support 4 million workers. This is extremely difficult.*

[Merchandiser, an RMG group]

Another representative highlighted the plight of RMG workers. Approximately 4 million workers are employed in both compliant and noncompliant factories, and their wages are directly tied to the factories’ income. Workers and individuals connected to the RMG industry will experience difficulties if these factories cannot sustain their operations. A management representative reinforced this sentiment:

*The entire garment sector is at risk. Even if some factories collapse, millions of families will be affected; only Allah (God) knows what will happen!*

[Assistant manager, a textile factory in Bangladesh]

## Discussion

### Principal Findings

We interviewed RMG workers and stakeholders from the RMG industry in Bangladesh. The interviews provided insights into the working environment, work experiences, perceptions of health care, lifestyle, and technology use, which varied depending on the type of RMG factory. This variation significantly impacted the well-being of the workers. Specifically, workers from compliant factories received essential health care support and demonstrated more effective use of technology than those in noncompliant factories. The disparities between compliant and noncompliant factory workers in our study mirror the regional disparities observed in labor market analyses [27]. As the pandemic spread globally, the circumstances for all workers changed, irrespective of their factory type. This shift profoundly affected their daily lives, raised health and safety concerns, and created a lack of emergency support.

The RMG industry faced numerous challenges, highlighting the urgent need for focused emergency relief and health care solutions for this community. Although the RMG industry has since begun to recover from the pandemic impacts, our findings reveal the community’s vulnerabilities during emergencies. Based on these insights, we proposed a technology-based framework design, VOICE, to improve the lifestyle and well-being of this community, which struggles with limited exposure to technology. This initiative could also benefit other low-income communities in the future.

### Requirement of Contextualized and Accessible Technology Intervention

The participants expressed that they work hard in a noncompliant work environment, but overall neglect their health issues. Although several earlier studies have highlighted the challenges faced by RMG workers. For instance, one study examined the workplace challenges faced by RMG workers in both export processing zone and nonexport processing zone factories [56,57]. While their findings were similar to those observed in noncompliant factories (theme 1), the studies did not address the conditions in compliant factories (addressed by theme 2), which is a crucial point to note, because compliant factory workers are facilitated with better support at the workplace. We cannot change the work environments of noncompliant factories, which depend on high-level stakeholders, and the National Tripartite Plan of Action on Fire Safety and Structural Integrity is constantly working to improve them [13,15,58]. We mostly consider their health problems, as previous studies found similar findings [21-24,59], but this worsened during the pandemic. They also needed emergency relief to survive. Another study analyzed the numerical impact of the COVID-19 pandemic on RMG workers [55] but did not empirically examine the situation (addressed by theme 3). Our research differs from existing research, focusing on supporting the health and emergency response of RMG workers through technological implications.

During the COVID-19 pandemic, workers lacked emergency aid, including health care support, financial assistance, and food. National policymakers prioritized monetary factors over workers’ safety and health. As a result, RMG factories remained open [60], leading to confusion over the payment of outstanding employee salaries and prompting large numbers of workers to return to their factories [61]. Thousands of RMG workers walked toward factories as public transport was unavailable due to lockdown measures [60]. This situation highlighted a gap in information dissemination and created confusion about where to seek help. The prolonged COVID-19 crisis led to the spread of the Delta variant, resulting in further factory closures [62] and the need for emergency support. Later, the Omicron variant also affected RMG factories [63]. The disparities experienced during this crisis called for urgent attention. The continuous struggle of the workers, who held on to hope for a better future, was evident in their dreams. Maintaining social distance [64] was frustrating for many, as they relied on daily earnings. Some relocated to villages to manage their expenses, disregarding the risks of COVID-19 [32,33,65]. Engaging participants enabled researchers to explore ways to help communities seek assistance and support from one another. The intuitive systems designed for Western countries require redesigning and reforming to better suit the conditions of poverty-stricken, densely populated regions like Bangladesh [66]. The challenges participants face present design opportunities to incorporate the voices of low-income and marginalized communities.

RMG workers in Bangladesh frequently struggled to access online health care services [38,67,68] due to limited access to and knowledge of technology. Toyama [69] discussed that technology integration could address existing societal problems; however, low-resource communities often require unique, simple, and effective systems that accommodate limited technology options. Connecting them with available technology poses challenges. Thus, a contextualized design approach can support users with restricted technological capabilities. That is why understanding their technological familiarity through this research was important.

Information from various contact points is widely available on social media but remains inaccessible to many communities. For instance, the recent flooding in southern Bangladesh demonstrated that obtaining information from victims was challenging, and many victims were excluded from emergency relief [70]. A point-of-contact–based service could function similarly to Facebook’s service in terms of location and crisis response during emergencies [34]. One notable example is Ushahidi, a crisis-mapping organization in Kenya, Afghanistan, Haiti, and Mexico that gathers data from social media networks [35,71]. In Bangladesh, OpenStreetMap has delivered emergency support to remote regions during natural disasters [36]. In Pakistan, experts have suggested that coordination platforms could be effective tools for facilitating communication among donors and volunteers during emergency relief [72].

During the COVID-19 crisis, nongovernmental organizations and foundations in Bangladesh collaborated on a single online platform to offer nationwide support [73]. For instance, to address food and medicine shortages among low-income and marginalized communities, Building Resources Across Communities developed a map pinpointing slum areas in Bangladesh, helping donors assist at specific locations [74]. Existing emergency response systems often require internet access and advanced technology, whereas a more feasible service could leverage mobile networks across Bangladesh, enabling communities to reach out via a toll-free number. Toll-free services have been implemented multiple times in Bangladesh [37,38,67,68,73], but these efforts have not yet converged to create a platform for donors and volunteers during emergencies [72]. Our understanding of RMG workers showed that they possess mobile phones but limited skills. Therefore, the technology design should consider a simple mechanism that requires minimal new skill learning.

### Design Implication as VOICE: Merging Service Interface and Service Providers

#### Overview

We propose a technology-based framework design named “VOICE,” focusing on consolidating existing support systems into a single minimalist platform, working as a community to assist low-income and digitally low-literate groups, particularly in emergency support and health care, using their existing technology access and knowledge. While advanced AI-based health assessment tools show promise, their deployment in low-resource settings requires careful attention to evidence of grounding and safety evaluation to prevent harm from unverifiable outputs [40]. Our VOICE framework avoids these complexities by providing a simple and human-mediated interface. Table 3 shows how the empirical findings guide the design considerations for VOICE. Then, in the VOICE framework design illustrated in Figure 2, a service interface will collaborate with service providers to offer user-requested services to communities and individuals, and the communication model will be virtual.

**Table 3.** Bridging empirical insights to the VOICE^a^ framework design.

Empirical finding	Design implication	VOICE feature
Workers have limited digital literacy and use basic phones (theme 1)	System must function without smartphone or internet dependency	Toll-free number as primary access point
Workers lack access to health care during emergencies (themes 2 and 3)	Need for integrated health support regardless of factory type	Health care provider integration module
Workers face sudden factory closures without communication (theme 3)	Need for emergency alert systems accessible to all	Broadcast notification capability
Workers live in congested areas with shared facilities (theme 3)	Support systems must account for community-level needs	Location-based relief coordination
Workers lack knowledge of available services (themes 1‐3)	Single, consolidated platform needed	Unified service interface aggregating existing providers
Workers experience financial constraints (theme 1)	Service must be free to access	Toll-free number with no call charges
Workers change locations or phone numbers frequently (themes 2 and 3)	System must accommodate mobile populations	Dynamic user database with flexible contact updates

a VOICE: Virtual Outlet for Integrated Community Engagement.

**Figure 2.**
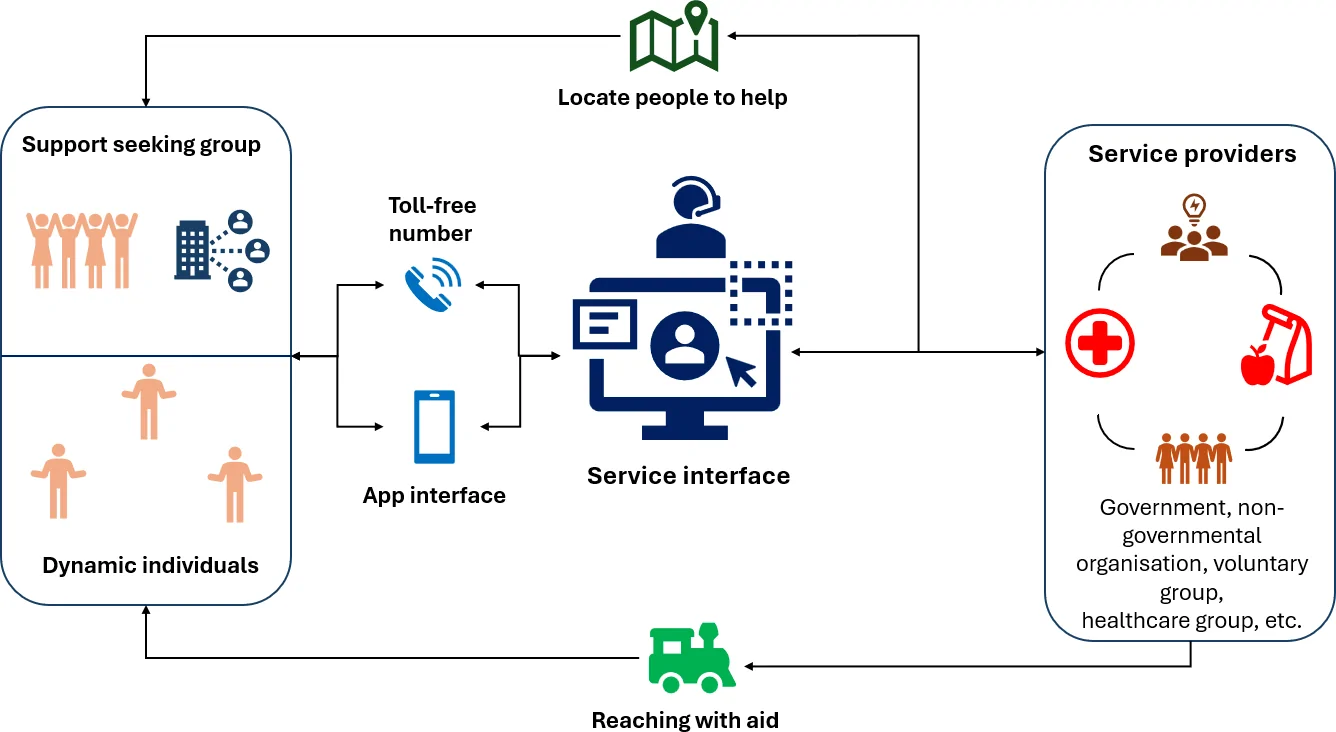
VOICE framework for supporting low-income groups. NGO: nongovernmental organization; VOICE: Virtual Outlet for Integrated Community Engagement.

#### Service Interface

The service interface will act as a platform that provides all users with a toll-free number, enabling multiple ways to access these services in their native language. This will facilitate interactions between service providers and individuals or groups seeking support. This will allow individuals to request assistance with emergency aid, health care, and related matters. For instance, if someone requests emergency assistance, the service interface can create a location-based database, as has been done in South America to map the supply chain of the offshore wind industry and send the main goods when demand is high [75]. This database can identify nearby individuals who may also need help, enabling efficient distribution of emergency relief. Similarly, if a group seeks health care assistance, the service interface can connect them remotely with a health care platform. At this point, the service interface can initiate the Relief Centre model suggested by Ozen and Krishnamurthy [76] to expand the services, which will include points of distribution, physical capacity such as volunteers, potential inventories to points of distribution, and a triage queue to check victim information before item distribution.

Even outside of crises, service providers can help individuals access the regular health care system at a marginal cost by using the databases available through the service interface. Though it is discussed that emergency medical services development in low-income countries involves understanding the unique needs of diverse communities, requires broad stakeholder involvement to maintain volunteer networks, and can be achieved through low-cost, bottom-up strategies [8], these needs need to be incorporated into the service interface and service providers. The community’s mobility is essential, as workers often change locations, contact points, and phone numbers over time. Therefore, community contact points must be flexible and able to adapt to real-time needs, given the dynamic nature of these populations. Stakeholders, such as telecommunications companies, can support and facilitate the service interface model.

#### Service Providers

We examine the existing service providers in Bangladesh that use various interfaces inspired by best practices. For example, mapping tools are designed to connect donors with needy communities [74]. Some nongovernmental organizations and volunteer organizations contacted low-income individuals to offer assistance [73]. The Bangladeshi government sold rice at BDT 10 per kilogram to support low-income families [77]. There are also health care platforms [38,67,68] that support individuals seeking help. However, many of these services currently use a top-down approach, in which providers conduct community needs assessments. In this proposed technology-based design, we suggest that service providers and interfaces collaborate on a single platform, thereby enhancing the effectiveness of the services offered. This VOICE framework design and its possibilities could ensure that the voices of low-income communities are heard, with implications for future developments.

#### Addressing Digital Literacy Barriers in VOICE Design

Our findings revealed that while 51% (28/55) of participants had smartphones (themes 1 and 2), actual use was limited to basic calling apps (eg, IMO), with few accessing health services online. This digital literacy gap informed 4 core design principles in VOICE:

- No internet dependency: Workers do not need internet connectivity or data plans. The system operates through standard cellular networks in the country.
- No cost to access: Users do not incur call charges, eliminating financial barriers. This was critical, given workers’ economic constraints (theme 1).
- Language accessibility: All interactions can be performed in their native language using simple, nontechnical vocabulary.
- Human backup: Live operators can be available 24/7 for users who cannot navigate automated systems, recognizing that some workers may need human assistance.

By focusing on these aspects, VOICE aims to make support accessible even to users who have never used digital services before.

### Limitations and Way Forward

We have acknowledged our limitations for this research. First, we interviewed 55 RMG workers before the COVID-19 pandemic, but we could reach only 12 during the pandemic. This reduction reflects the unprecedented disruption caused by Bangladesh’s national lockdown in April 2020, including transportation suspension, worker displacement, and communication barriers. This limited participation limits us from gaining insight into all the unique challenges the pandemic poses. The findings from phase 2 are presented as exploratory insights rather than statistically representative data. Similar to qualitative research, the focus is on depth and theoretical saturation rather than sample size. The data from phase 2 provided a rich and contextual understanding of the immediate impact of the pandemic on a specific group of workers. In addition, such limitations can potentially be mitigated in future emergencies through the following recruiting strategies by Kim et al [78], who recruited rural participants during the COVID-19 pandemic via local pharmacist referrals and community outreach, which helped facilitate communication and build trust and rapport between participants and researchers.

Second, during FGDs, not all participants were equally responsive because we had to arrange large groups, leading a few to remain silent. According to Simmel’s [79] theory of group size, large groups result in weaker member involvement and participation. At this point, we noted nonverbal cues (eg, expressions) and brief comments from quieter participants, which were included in the analysis. Silent participants’ perspectives were also partly captured through the similarities in experiences expressed by more vocal participants and the consistency of themes across groups. In future research, it is necessary to arrange small focus groups to obtain rich data, primarily from female participants [80]. In addition, social, religious, and aristocratic sectors are relevant for small interviews [79].

Third, following this study, it took approximately 3 years to achieve regular support; however, the necessary support systems during the emergency were not implemented. In future research, we can involve such RMG communities in design sessions, such as co-design or participatory design workshops, where users can cocreate or ideate the design recommendations, highlighting the practical real-world challenges and making the system inclusive [81,82].

### Conclusions

This research explored the workplace environment, daily lives, challenges, personal perspectives on health care, and the use of technology by RMG workers, as well as the crisis they faced during the pandemic. We found that many of them neglect their health care needs and require more support from various sources. The complexity of issues arising from the COVID-19 pandemic is significant. The workers expressed significant concerns about job security, food availability, and health care, highlighting the gaps in support that are often overlooked by authorities and themselves. The workers’ distress was echoed by the stakeholders. In response, we have proposed a technology-based conceptual framework, VOICE, that suggests workers could connect with service providers through a simple point-of-contact interface, such as a toll-free number. This setup would enable service providers to reach marginalized communities during emergencies or in regular situations, providing the support needed to improve their lives and well-being.

## Abbreviations

FGD: focus group discussion
KI: key informant
RMG: ready-made garment
VOICE: Virtual Outlet for Integrated Community Engagement
